# Inferring Phylogeny and Introgression using RADseq Data: An Example from Flowering Plants (*Pedicularis*: Orobanchaceae)

**DOI:** 10.1093/sysbio/syt032

**Published:** 2013-06-14

**Authors:** Deren A. R. Eaton, Richard H. Ree

**Affiliations:** ^1^Committee on Evolutionary Biology, University of Chicago, Chicago, IL 60637, USA; and ^2^Botany Department, Field Museum of Natural History, Chicago, IL 60605, USA

## Abstract

Phylogenetic relationships among recently diverged species are often difficult to resolve due to insufficient phylogenetic signal in available markers and/or conflict among gene trees. Here we explore the use of reduced-representation genome sequencing, specifically in the form of restriction-site associated DNA (RAD), for phylogenetic inference and the detection of ancestral hybridization in non-model organisms. As a case study, we investigate *Pedicularis* section *Cyathophora*, a systematically recalcitrant clade of flowering plants in the broomrape family (Orobanchaceae). Two methods of phylogenetic inference, maximum likelihood and Bayesian concordance, were applied to data sets that included as many as 40,000 RAD loci. Both methods yielded similar topologies that included two major clades: a “rex-thamnophila” clade, composed of two species and several subspecies with relatively low floral diversity, and geographically widespread distributions at lower elevations, and a “superba” clade, composed of three species characterized by relatively high floral diversity and isolated geographic distributions at higher elevations. Levels of molecular divergence between subspecies in the rex-thamnophila clade are similar to those between species in the superba clade. Using Patterson’s D-statistic test, including a novel extension of the method that enables finer-grained resolution of introgression among multiple candidate taxa by removing the effect of their shared ancestry, we detect significant introgression among nearly all taxa in the rex-thamnophila clade, but not between clades or among taxa within the superba clade. These results suggest an important role for geographic isolation in the emergence of species barriers, by facilitating local adaptation and differentiation in the absence of homogenizing gene flow. [Concordance factors; genotyping-by-sequencing; hybridization; partitioned D-statistic test; *Pedicularis*; restriction-site associated DNA.]

A general problem in phylogeny reconstruction is the difficulty of resolving relationships among closely related, recently diverged lineages (Maddison and Knowles 2006). Part of the difficulty is practical, as commonly sequenced genetic markers often lack sufficient phylogenetic signal at the lowest taxonomic levels. More fundamentally, however, biological processes such as incomplete lineage sorting and horizontal gene transfer create conflict among gene trees, complicating the task of inferring phylogeny as a single bifurcating history (Maddison 1997). In this context, incomplete lineage sorting can be thought of as statistical noise, arising from stochastic coalescence, that can mask the signatures of true phylogenetic divergence events. Horizontal gene transfer, on the other hand, represents lineage reticulation, both in the form of introgressive gene flow and hybrid speciation. Both processes might be expected to have higher prevalence between closely related species and populations that have incomplete reproductive isolation.

Attempts to overcome the phylogenetic challenges posed by both incomplete lineage sorting and horizontal gene transfer are represented by much recent work on multi-locus methods for inferring species trees (or networks) based on gene tree coalescence (e.g., Ané et al. 2007; Liu 2008; Kubatko 2009; Kubatko et al. 2009; Yu et al. 2011). These methods rely on the availability of multiple unlinked nuclear markers with levels of sequence variation appropriate for the phylogenetic scale of inquiry. For recent divergence events in non-model organisms, obtaining such multigene data sets remains challenging, because even if a sufficient number of orthologous nuclear loci can be identified and amplified reliably, individual genes are less likely over shorter time scales to accumulate variation sufficient for resolving informative gene trees (e.g., O’Meara 2010).

High-throughput sequencing technologies now offer much greater potential for efficiently sampling entire genomes of any taxon for phylogenetically informative variation. In particular, reduced-representation methods (reviewed in Davey and Blaxter 2010), such as restriction-site associated DNA sequencing (RADseq; Miller et al. 2007; Baird et al. 2008; Rowe et al. 2011), or genotyping-by-sequencing (GBS; Elshire et al. 2011), allow the regions adjacent to restriction sites to be surveyed with deep coverage at a high ratio of samples to sequencing effort. These methods are particularly appealing for systematics research because they are easily applied to non-model organisms for which no reference genome sequence is available. In contrast to data sets for gene tree analysis in which sequence lengths are relatively long but the number of sampled genes is relatively small, RADseq generates data sets of relatively short sequences from a large number of loci. The potential for informative gene tree variation within a single RAD locus is thus relatively small, but to the extent that restriction sites are conserved across samples, the collective potential of RADseq for detecting many single-nucleotide polymorphisms (SNPs) across the genome is large. For this reason, it has been applied to date almost exclusively to population-level studies (Baird et al. 2008; Emerson et al. 2010; Baxter et al. 2011; Hohenlohe et al. 2011; Pfender et al. 2011), although attention is now turning to its utility for phylogenetics (e.g., Rubin et al. 2012).

RADseq data sets are attractive for systematic studies of closely related lineages because they offer the potential of detecting current or historical introgression, even at very low levels (Twyford and Ennos 2011). In particular, Patterson’s D-statistic test (Durand et al. 2011) is designed to detect ancient admixture (hybridization) between diverged lineages based on the frequencies of discordant SNP genealogies in a pectinate four-taxon tree. The test was notably used to infer interbreeding between ancestral populations of modern humans and Neanderthals outside of Africa (Green et al. 2010). With sufficiently large samples of markers from across the genome provided by RADseq data, the test can be similarly applied to clades of non-model species as a test for post-divergence gene flow between close relatives.

In this article, we investigate the utility of RADseq data for resolving recalcitrant phylogenetic relationships in the angiosperm genus *Pedicularis* (Orobanchaceae, the broomrape family), which consists of about 700 species of hemiparasitic herbs having a center of diversity in the Hengduan Mountains of south-central China (Yang et al. 1998). *Pedicularis* is known for exhibiting spectacular interspecific variation in floral traits that is thought to reflect adaptations to reduce heterospecific pollen flow by generalist bumble bees (Macior 1983; Grant 1994; Eaton et al. 2012). We focus our attention on *Pedicularis* sect. *Cyathophora*, a clade of five described species (*P. cyathophylla*, *P. cyathophylloides*, *P. rex*, *P. superba*, and *P. thamnophila*) that are all endemic to the Hengduan region. This clade, which is easily recognized by the distinctive fusion of leaves around the stem, is particularly interesting in the context of floral homoplasy, as its species collectively exhibit almost the full range of floral variation found across the genus as a whole (Fig. 1). Within it, *P. rex* exhibits the most intraspecific variation, with several described subspecies and varieties that vary not only in floral shape and size but also color. Resolving the phylogeny of *Pedicularis* sect. *Cyathophora* thus has the potential to yield insights into the diversification of flowers and lineages at a relatively fine scale that may illuminate general evolutionary patterns across the genus as a whole.

**Figure 1:**
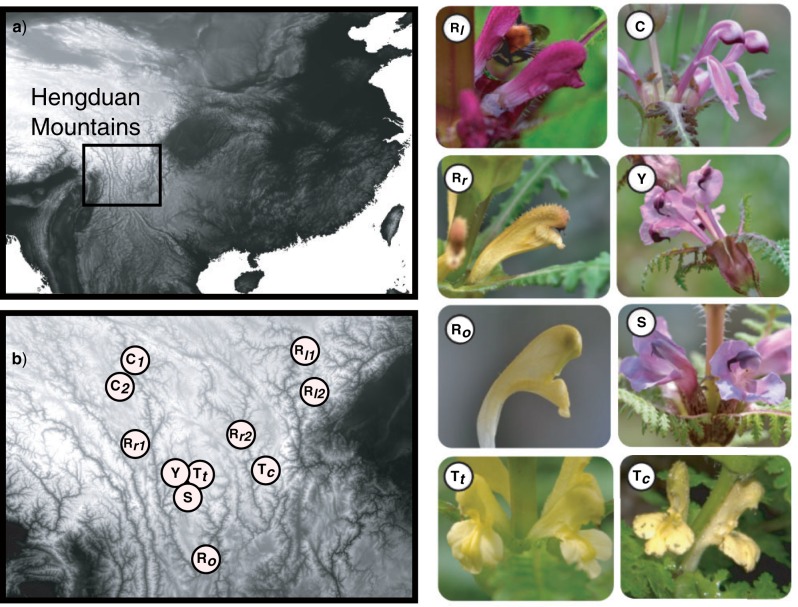
Sampling localities and photographs of taxa in *Pedicularis* sect. *Cyathophora*. Codes for taxon names are listed in Table 1.

Previous phylogenetic analyses of a nuclear marker (ITS, the internal transcribed spacer region of nrDNA) and a chloroplast marker (*matK*) strongly supported the monophyly of *Pedicularis* sect. *Cyathophora*, but provided little phylogenetic resolution within it, and showed intergenomic conflict in the position of *P. cyathophylla* (Ree 2005). These results raise the question of whether phylogenetic relationships have been obscured by gene flow between species. Little is known about hybridization in *Pedicularis*: putative F1 hybrids have rarely been found in nature, no hybrid species have been described, and the only experimental interspecific cross reported in the literature, between *P. longiflora* and *P. rhinanthoides*, showed heterospecific pollen tube growth but no seed set (Yang et al. 2007). However, it is also the case that no crossing experiments have been reported for closely related species such as those in *P.* sect. *Cyathophora*. Moreover, other factors suggest at least the potential for interspecific gene flow. In the Hengduan region, the many species of *Pedicularis* frequently co-occur in sympatry, flower synchronously during the short temperate-alpine summer, and share bumble bee pollinators. The Hengduan species are also uniformly diploid; chromosome counts show sporophytic values of 2*N* = 16, and no species are reported as polyploids or aneuploids (Goldblatt and Johnson 2010), suggesting a lack of karyotypic barriers to interbreeding.

Here, we investigate the utility of RADseq data for inferring phylogeny and historical introgression in *Pedicularis* sect. *Cyathophora*. As phylogenetic workflows for processing RADseq data are not yet standard, we include a description of our software pipeline for clustering, filtering, and sorting RAD sequences into phylogenetically informative alignments of putative loci. We then apply three different methods for reconstructing the phylogeny of *P.* sect. *Cyathophora* using RADseq data. To test for introgressive gene flow between lineages, we apply the D-statistic to various four-taxon subtrees, and present a novel extension of the D-statistic which allows investigation of the temporal sequence of multiple introgression events. We show how this new method can be used to infer phylogenetic relationships that are otherwise obscured by gene flow, demonstrating this with both a simulated data set as well as our empirical data set. We discuss the results in the context of how floral variation and geographic ranges may have influenced propensities for hybridization and speciation during the diversification of *P.* sect. *Cyathophora*.

## Materials and Methods

### Taxon Sampling

Genomic DNA was extracted from silica-dried leaf tissues of voucher specimens collected between 2007 and 2009 in Yunnan, Sichuan, and Xizang (Tibet), China (Fig. 1). Thirteen samples represent the five species in *Pedicularis* sect. *Cyathophora* as well as the closest known outgroup species, *P. przewalskii*. The ingroup samples include one individual of *P. cyathophylla*, one of *P. superba*, two of *P. cyathophylloides*, two of *P. thamnophila* representing the subspecies *P. thamnophila* subsp. *cupuliformis* and *P. thamnophila* subsp. *thamnophila*, respectively, and five of the geographically widespread species *P. rex*. The latter includes two individuals of *P. rex* subsp. *lipskyana*, two of *P. rex* subsp. *rex*, and one of *P. rex* subsp. *rex* var. *rockii*. For convenience, we refer to this last individual informally in this article as *P. rex* subsp. *rockii*. Voucher information is available in online Appendix 1 (doi:10.5061/dryad.bn281).

### RADseq Data Acquisition and Analysis

Library preparation and sequencing of RAD markers from genomic DNAs was performed by Floragenex Inc. (Eugene, Oregon) using the restriction enzyme *Pst1* and sample-specific barcodes. The 13 samples studied here were pooled with 11 others and run multiplexed on a single lane of an Illumina GAIIx sequencer for 75 cycles to generate single-end reads.

To process the raw RADseq data (Illumina FASTQ output files) for phylogenetic analysis, we developed a custom software pipeline for distinguishing sequencing errors from nucleotide polymorphisms within samples, identifying putative orthology relationships across samples, and assembling formatted data files. This pipeline, called *pyRAD* (http://pyrad.googlecode.com), is somewhat different from other RADseq software packages (e.g., *Stacks*; Catchen et al. 2011) that emphasize analysis of population-level variation. Because the objective of *pyRAD* is to capture variation across species and potentially higher-level clades, we employ a global clustering/alignment method, in contrast to the “off-by-N” approach of *Stacks*. This allows our method to cluster sequences with higher levels of divergence, including indel variation. D-statistic tests are also performed in *pyRAD*. The following sections describe the *pyRAD* pipeline in more detail.

*Preparing sequence files for analysis.—*Given one or more Illumina sequence files in FASTQ format, *pyRAD* can de-multiplex the data and create separate files for each sample. Sequences are identified allowing for one base mismatch in their sample-specific barcode. The restriction site and barcode are trimmed from each sequence, and bases with a FASTQ quality score below a given value (here, 20) are replaced with *N*. Sequences having more than a given percentage of *N*s (here, 5%) are discarded.

*Clustering RAD sequences within samples of genomic DNA.—*For each sample, sequences are clustered by similarity (here, 90%) using the *uclust* function in USEARCH (Edgar 2010) with heuristics turned off, yielding clusters representing putative loci. Clusters of fewer sequences than a set minimum depth of coverage (here, 6) are excluded in order to ensure accurate base calls. The remaining clusters can then either be exported to external genotyping software or processed within *pyRAD* to generate consensus sequences. In *pyRAD*, the error rate (∈) and heterozygosity (*π*) are jointly estimated from the observed base counts across all sites in all clusters, by applying the maximum-likelihood equation of (Lynch 2008). The mean ∈ is then used to assign consensus diploid genotypes for each site in each cluster by calculating the binomial probability the site is homozygous (aa or bb) versus heterozygous (ab) given the relative frequencies of observed bases at the site and ∈ (Li et al. 2008). If a base cannot be assigned with ≥ 95% probability it is replaced by *N* in the consensus sequence. Heterozygotic variation is recorded using appropriate ambiguity codes. The end result of this step is a set of consensus sequences of putative RAD loci for each barcoded DNA sample.

*Clustering and filtering RAD loci across samples—*Consensus sequences from all samples are clustered by sequence similarity, with their input order randomized, using the same similarity threshold as in the previous step of within-sample clustering. The resulting clusters representing putative RAD loci shared across samples are then aligned with Muscle (Edgar 2004). Any locus appearing heterozygous at the same site across a set number of samples (here, 3) is discarded as likely representing a clustering of paralogs, under the assumption that paralogs are more likely than ancestral polymorphisms to be shared across multiple species or samples (Hohenlohe et al. 2011).

The remaining clusters are treated as RAD loci, that is, multiple alignments of putatively orthologous sequences, and are assembled into phylogenetic data matrices. For any given RAD locus, sequences of one or more samples may be missing if substitutions in the restriction site have disrupted recognition, or if the locus did not receive sufficient coverage for confident basecalling. To explore the effect of missing data, we compiled two supermatrices that differed in their amounts of missing data: a “minimum-taxa” data set containing all loci for which more than four samples were present, and a “full-taxa” data set containing only loci for which all 11 ingroup samples were present.

### Simulation of RADseq Data Sets

**Figure 2:**
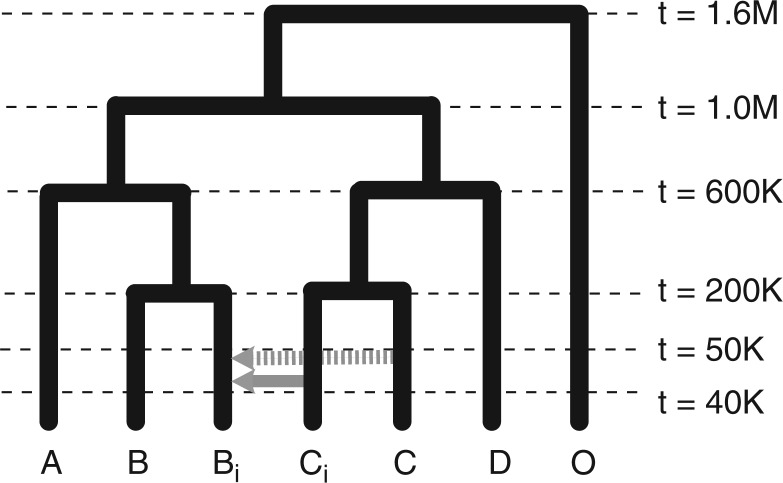
Coalescent model used to simulate RADseq-like data. Unidirectional migration (gene flow) from taxon C*_i_* into B*_i_* (solid gray arrow) occurred over 10,000 generations, beginning 50,000 generations in the past. Three data sets were created with different rates of gene flow, and one additional data set with gene flow from both C and C*_i_* into B*_i_* (dashed gray arrow).

For the purpose of validating our analyses of phylogeny and introgression in *Pedicularis* sect. *Cyathophora*, we simulated RADseq-like data under a coalescent model with varying degrees of interspecific gene flow. These were implemented in Python using the EggLib library (Mita and Siol 2012). Sequences were evolved on a phylogenetic tree of seven species (Fig. 2) to create 30,000 loci, each 200 bp in length, using parameter values intended to reflect the clade of herbaceous plants under study, including the *Arabidopsis* mutation rate of 7×10^−9^ (Ossowski et al. 2010) and effective population size *N* = 100,000. These values were held uniform across taxa (Θ = 4*Nμ* = 0.0028). In one set of simulations, unidirectional gene flow occurred from one taxon into another (C*_i_* and B*_i_*, respectively; Fig. 2) over a period of 10,000 generations, beginning 50,000 generations before the present. We created three data sets varying in the strength of introgression (probability of migration between taxa), with rates equal to 10 migrants per generation, 1 migrant per generation, and 1 migrant per 10 generations. We refer to these as the strong, medium, and weak gene flow data sets. In addition, a “multiple-reticulation” data set was created by simulating a scenario in which two sister taxa (C*_i_* and C) both introgress into B*_i_* independently over the same 10,000 generation period, at a rate equal to that of the weak gene flow simulations.

### Phylogenetic Inference

To infer phylogeny from the empirical and simulated RADseq data sets described above, we first applied a supermatrix approach in which all RAD loci were concatenated into a single alignment, with missing data (Ns) entered as needed for loci with incomplete taxon sampling (de Queiroz and Gatesy 2007). Maximum-likelihood trees were inferred for the minimum-taxa and full-taxa data sets using RAxML 7.2.8 (Stamatakis 2006), with bootstrap support estimated from 200 replicate searches with random starting trees using the GTR+Γ nucleotide substitution model.

We also applied Bayesian concordance analysis (Baum 2007) using BUCKy 1.4.0 (Larget et al. 2010). Unlike multispecies coalescent methods, concordance analysis is applicable to cases where gene tree incongruence is caused by a combination of incomplete lineage sorting and reticulate evolution (hybridization and introgression) (Baum 2007; Ané et al. 2007). It provides estimates of concordance factors (CFs) that measure the proportion of genes for which a clade is true. These can be summarized by a primary concordance tree that is composed of clades having CFs greater than any contradictory clade. BUCKy also infers a population tree that is expected to converge on the true tree when all discordance is caused by incomplete lineage sorting. Comparing a primary concordance tree and a population tree can thus potentially provide insight into the influence of incomplete lineage sorting versus introgression.

BUCKy takes as input a posterior sample of gene trees estimated for each individual locus. For this purpose, we selected loci from the full-taxa data set that contained at least two phylogenetically informative SNPs, excluding sequences of the two outgroup taxa (*Pedicularis przewalskii*) and redundant individuals representing *P. cyathophylloides* and *P. rex* subsp. *lipskyana*. For each locus, we executed two independent runs of MrBayes 3.2.1 (Ronquist et al. 2012) using the GTR+Γ substitution model, each run with four chains for 1,010,000 generations. These sampled a total of 2,200 trees from the posterior distribution per locus, of which the first 200 were discarded as burn-in. We ran BUCKy with four chains for 500,000 generations at two different values of *α*, the prior on the number of unique gene tree topologies: 0.1 and 100.

### Tests for Introgression

**FIGURE 3:**
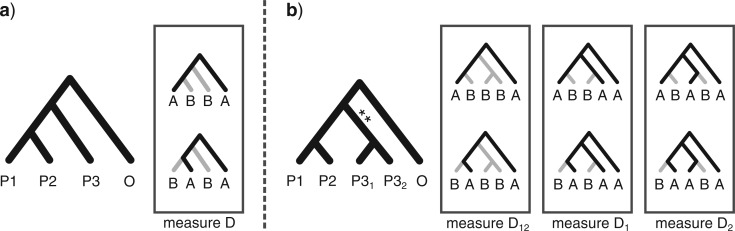
A schematic description of Patterson’s four-taxon D-statistic test for hybridization and the five-taxon partitioned D-statistic test. a) The four-taxon test measures asymmetry in the occurrence of two incongruent allele patterns (shown in the box), which are expected to arise with equal frequencies in the absence of introgression, but to deviate from symmetry if P3 exchanged genes with either P1 or P2 to the exclusion of the other. b) The five-taxon test samples an additional population within the P3 lineage, the two samples now denoted P3_1_ and P3_2_, and measures asymmetry in three sets of allele patterns (the three boxes shown), thus measuring three separate D-statistics. The first (D_1_) measures asymmetry in incongruent alleles where the derived allele is present in P3_1_ but not P3_2_, the second (D_2_) where it is present in P3_2_ but not P3_1_, and the third where the derived allele is shared by both P3_1_ and P3_2_, having arisen in their ancestor (branch indicated by **).

*Four-taxon D-statistic test.—*We used the D-statistic (Green et al. 2010; Durand et al. 2011) to test whether introgression had occurred between two lineages in a given phylogeny. From a pectinate topology (((P1,P2),P3),O), the genome-wide frequencies at which two incongruent allele patterns appear across the tips (ABBA and BABA) can be used to infer hybridization (Fig. 3a). These patterns, in which the taxon P3 exhibits a derived allele relative to the outgroup O that is shared only by P1 or P2 (but not both), represent gene histories that are incongruent with the phylogeny. If the incongruence is caused by stochastic lineage sorting, the frequencies of ABBA and BABA are expected to be equal. Alternatively, if the cause of incongruence is introgression between P3 and either P1 or P2, the two patterns are not expected to occur with equal frequency. The D-statistic quantifies this asymmetry:

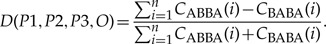

Here, *C*_ABBA_(*i*) is the number of SNPs showing the ABBA pattern in a given locus *i*, and the counts are summed over all loci. Conservatively, we excluded sites from this test which appeared heterozygous for any individual. To the extent that RAD sequences are a random sample of the genome, the D-statistic represents a genome-wide measure of introgression.

We used the D-statistic to test whether hybridization occurred within the rex-thamnophila clade, within the superba clade, and between these clades. In this context we refer to a “test” as the measurement of the D-statistic from a distinct four-taxon subtree extracted from a complete tree estimated in the previous step, fitting a pectinate topology (((P1,P2),P3),O) where each tip represents a species or subspecies. Each test in this sense can potentially sample different individuals representing the same terminal taxon, so we define a “replicate” as measurement of the D-statistic from one out of all possible combinations of individuals that could be sampled for a given subtree.

For each replicate, we ran 1,000 bootstrap iterations to measure the standard deviation of the D-statistic, in which loci were re-sampled with replacement to the same number as in the original data set. We report the results as the range of *Z*-scores across all replicates in a test, where *Z* is the number of standard deviations from 0 (the expected value) for D. Significance was assessed for each replicate by converting the *Z*-score into a two-tailed *p*-value, and using *α* = 0.01 as a conservative cutoff for significance after correcting for multiple comparisons using Holm–Bonferroni correction.

For tests showing significant introgression, we employed the equation of (Durand et al. 2011) to estimate the proportion of genomic introgression between taxa. This divides the numerator of the D-statistic test by the numerator of an alternative test measuring the maximum expected amount of introgression; in other words, introgression from P3 into a close relative from the same clade [e.g., (((P1,P3_1_),P3_2_),O)].

*Partitioned D-statistic test.—*The D-statistic as described above does not take full advantage of the information available from incongruent allele patterns in multiple taxa. Importantly, it detects only whether alleles from one lineage occur excessively in another lineage, but does not distinguish whether this stems from direct gene flow from the lineage in question, or gene flow from a close relative. This distinction becomes increasingly important when the D-statistic is applied at deeper or broader phylogenetic scales with redundant sampling of taxa. To demonstrate this problem, consider a case in which the P3 lineage comprises sister taxa P3_1_ and P3_2_, and that only P3_1_ is sampled (Fig. 3b). If the unsampled taxon P3_2_ hybridized with P2, but P3_1_ did not, the D-statistic may falsely indicate that introgression occurred between P3_1_ and P2. This is a consequence of the fact that alleles which introgressed from P3_2_ into P2 may also be shared between P3_2_ and P3_1_ due to their common ancestry exclusive of P2. In order to distinguish these possibilities, we developed a novel extension of the D-statistic, which we call the partitioned D-statistic test.

The partitioned D-statistic is applied as follows when two lineages from within the P3 clade have been sampled. These lineages, P3_1_ and P3_2_, are assumed not to have exchanged genes with each other. As before, we measure asymmetry in the counts of incongruent sites, with the key difference being that we now ask whether a derived allele is present in only P3_1_ and not P3_2_, in P3_2_ and not P3_1_, or whether it is shared by both (Fig. 3b). This amounts to measuring three D-statistics, one for each scenario, which we denote D_1_, D_2_, and D_12_, respectively. These are calculated in the same manner as the original D-statistic, but are based on five-taxon allele patterns. For example, for the species tree topology (((P1,P2),(P3_1_,P3_2_)),O), D_1_ takes as input the counts of ABBAA and BABAA, and measures the signal of introgression involving P3_1_, whereas D_12_ takes as input the counts of ABBBA and BABBA, and measures the signal of introgression involving the branch subtending the most recent common ancestor of P3_1_ and P3_2_.

To verify that the partitioned D-statistic can indeed distinguish single or multiple introgression events from the signal of shared ancestry, we applied it to two simulated data sets: the weak gene flow data set and the multiple-reticulation data set, which differ in whether one (C*_i_*) or two (C*_i_* and C) taxa are the source of introgression into another taxon (B*_i_*).

*Directionality of gene flow.—*While the four-taxon D-statistic cannot distinguish the directionality of gene flow, that is, whether it occurred from P2 into P3, from P3 into P2, or in both directions, the partitioned D-statistic can infer directionality through its measurement of introgression of shared ancestral alleles, D_12_. For example, if gene flow occurred from P3_1_ into P2, then derived P3 alleles which arose in the ancestor of P3_1_ and P3_2_, and are thus shared by both taxa, will also appear in P2. In contrast, if gene flow occurred only in the opposite direction, from P2 into P3_1_, P2 will not contain alleles that are shared by the two P3 taxa, and thus the partitioned test would find a non-significant D_12_. In this way, D_12_ acts as an indicator, showing whether introgression occurred from the P3 lineage into P2, versus whether the signal is caused by gene flow in the opposite direction. Contrast this with the four-taxon test, where a significant D for tests (P1, P2, P3_1_, O), (P1, P2, P3_2_, O), or (P3_1_, P3_2_, P2, O) would all indicate introgression, but fail to distinguish that only P3_1_ and not P3_2_ introgressed into P2 (which D_1_ vs. D_2_ would indicate), or that introgression occurred in only one direction (which D_12_ indicates). This is demonstrated in the Results section using the simulated data sets.

### Detecting Errors in Total-Evidence Tree Reconstruction Due to Introgression

*Partitioned D-statistic.—*By separating the signal of introgression into components attributable to shared versus independent ancestry, the partitioned D-statistic may also be used to test whether P3_1_ and P3_2_ are indeed monophyletic relative to P1, P2, and O. The reasoning is that if P3_1_ and P3_2_ are paraphyletic with respect to P1 and P2, as in the case of ((((P1, P2), P3_1_), P3_2_), O), then D_12_ will not deviate significantly from 0, because the P3 taxa do not share any common history independent of P1 and P2. We applied the partitioned D-statistic test in this way to investigate the relationship of the two subspecies of *P. thamnophila* with respect to the subspecies of *P. rex*.

*Censored comparisons of alternative topologies.—*We used the Shimodaira–Hasegawa test (SH test; Shimodaira and Hasegawa 1999) as implemented in RAxML to test whether total-evidence reconstructions of phylogeny might be positively misled by introgression, for example, if P3_1_ and P3_2_ are in fact sister taxa but appear paraphyletic because one (e.g., P3_1_) has introgressed with another lineage (e.g., P2). The procedure first applies the SH test to topologies with full taxon sampling, comparing a topology in which the taxa of interest are paraphyletic with a topology that differs only in having them monophyletic. The SH test is then repeated after removing from the data set one of the taxa suspected to have undergone introgression (e.g., P2). SH tests are thus always made between trees containing the same set of taxa. The rationale is that when one of a pair of reticulating taxa is removed from the analysis, introgressed sites which would otherwise be treated as synapomorphies (shared by the taxa, with the effect of pulling them erroneously together) will instead appear as autapomorphies in the remaining taxon, erasing the effect of introgression on the inferred topology. In other words, the objective is to “censor” the effect of introgressed DNA on phylogenetic inference.

To investigate the monophyly of *P. thamnophila*, we first applied the SH test before and after removing all samples of *P. rex* other than *P. rex* subsp. *rockii*. Then, we applied the SH test to compare the relationships among the three subspecies of *P. rex* before and after removing each subspecies of *P. thamnophila*.

## Results

### RAD Data and Processing

Illumina sequencing returned an average of 1.35×10^6^ reads per sample, which after filtering and clustering was reduced to an average of 48,000 clusters (or “stacks”, following the terminology of Emerson et al. 2010) with coverage greater than our set minimum of six, yielding a mean coverage depth of 19.80. Consensus sequences were called for each cluster, yielding approximately 45,000 loci per sample (Table 1). The ML estimate of the sequencing error rate is lower than heterozygosity (∈ = 2.3×10^−3^ and *H* = 6.7 ×10^−2^, respectively), and both values are within the range where simulation showed they could be accurately estimated (results not shown). The last five bases were trimmed from all loci, as the error rate was found to increase precipitously in this region, giving a final average read length of 65 bp.

**Table 1:** Results of filtering and clustering RAD sequences from 13 individuals of *Pedicularis*, identified in subsequent tables by codes in the ID column

Taxon	ID	RAD tags (×10^6^)	Clusters at 90%^a^	Mean depth	Consensus loci^b^	Minimum-taxa data set	Full-taxa data set
*P. rex* subsp. *rex*	R*_r_*_1_	1.71	54,832	24.45	51,525	35,021	4869
*P. rex* subsp. *rex*	R*_r_*_2_	1.41	54,220	20.11	49,556	33,991	4869
*P. rex* subsp. *lipskyana*	R*_l_*_1_	1.39	51,754	22.53	48,962	34,873	4869
*P. rex* subsp. *lipskyana*	R*_l_*_2_	0.82	41,576	13.95	38,653	28,351	4869
*P. rex* subsp. *rockii*	R*_o_*	1.80	53,135	21.68	50,020	34,313	4869
*P. thamnophila* subsp. *thamnophila*	T*_t_*	1.45	51,146	21.57	47,052	32,791	4869
*P. thamnophila* subsp. *cupuliformis*	T*_c_*	0.64	27,555	12.13	25,215	18,054	4869
*P. cyathophylloides*	C_1_	2.20	53,959	34.61	51,258	31,559	4869
*P. cyathophylloides*	C_2_	2.20	73,880	22.59	69,517	29,297	4869
*P. cyathophylla*	Y	1.25	50,357	16.37	46,510	26,053	4869
*P. superba*	S	0.70	32,970	14.16	30,628	20,726	4869
*P. przewalskii*	W_1_	0.96	39,621	17.00	36,231	12,244	2631
*P. przewalskii*	W_2_	1.00	44,207	16.25	40,670	14,288	2993

Note: The number of loci (clusters) having each sample in the minimum-taxa and full-taxa data sets are shown.

^a^Clusters with more than the minimum depth of five reads.

^b^Consensus loci which passed filtering for paralogs.

Clustering of consensus sequences across all 13 samples revealed 268,901 unique clusters. The minimum-taxa data set (loci with at least four samples) contained 42,235 loci and a total of 61,829 phylogenetically informative sites. The full-taxa data set (all loci have complete sampling of the 11 ingroup individuals) contained 4,837 loci and 8,227 phylogenetically informative sites. The proportion of missing data in each case was 37% and 7.6%, respectively. The occurrence of each sample in the final data sets was relatively uniform, with the outgroup samples being recovered least often (Table 1). The BUCKy data set included 945 loci containing at least two phylogenetically informative sites among the nine selected ingroup samples.

### Phylogeny Reconstruction

The ML, primary concordance, and population trees for *Pedicularis* sect. *Cyathophora* were all congruent with the ITS topology of (Ree 2005) in recovering the rex-thamnophila and superba clades, with *Pedicularis cyathophylla* nested within the latter. Resolution within clades was less certain, as described below. From simulated data, the correct topology was recovered for the low and medium gene flow data sets, but not in the strong gene flow data set.

**FIGURE 4:**
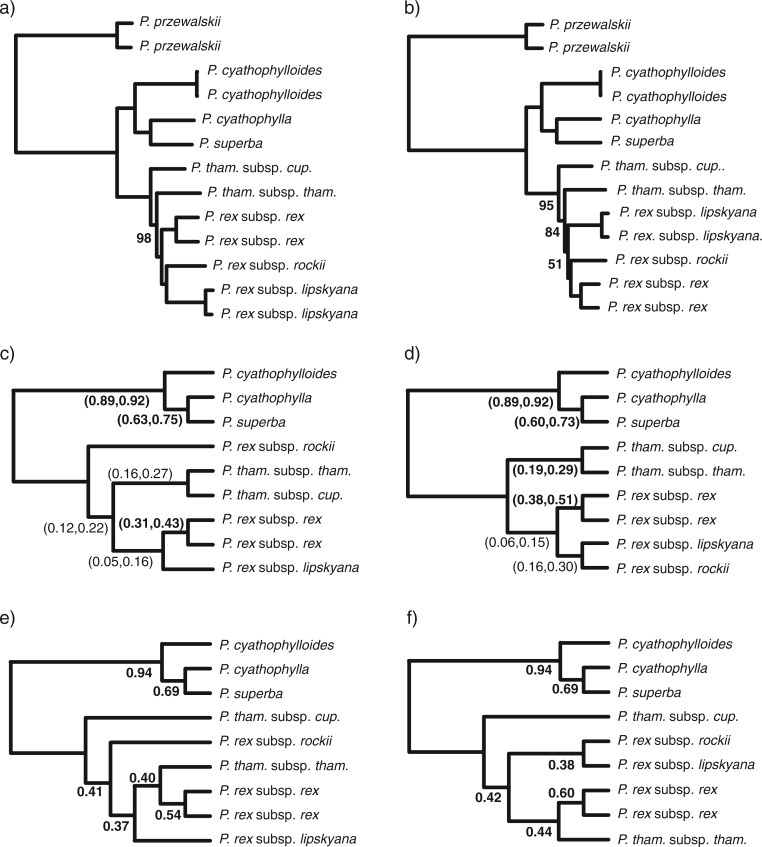
Phylogeny of *Pedicularis* sect. *Cyathophora* inferred from RADseq data. ML trees were estimated from the sparse (minimum-taxa) supermatrix a) and the densely sampled (full-taxa) supermatrix b), yielding high bootstrap support (100 except where indicated). Primary concordance trees c, d) and population trees e, f) were inferred on 945 variable loci from the full-taxa supermatrix, at either *α* = 0.01 (c, e) or 100 (d, f). The 95% CI for CFs are shown on primary concordance trees, those in bold did not overlap with any conflicting CF. Quartet CFs are shown on population trees.

ML analysis of the minimum-taxa (sparse) and full-taxa (dense) supermatrices yielded topologies with comparable branch lengths, but which differed in their resolution of the three subspecies of *P. rex* (Fig. 4a,b). In both trees, the subspecies of *P. thamnophila* were paraphyletic. The minimum-taxa data set gave high bootstrap support for all clades, whereas the full-taxa tree had lower support for shorter branches. For all the simulated data sets, including the strong gene flow data set, inferred ML trees had uniform bootstrap support of 100% for all branches.

For the Bayesian concordance analysis we report CFs as their mean and 95% confidence intervals (CI) on the primary concordance trees, and as the quartet CF for population trees. CFs in the primary concordance tree that do not overlap in their CI with any conflicting clade are considered significantly supported. A full account of the BUCKy analyses is provided in online Appendix 2.

Concordance factors provided an accurate measure of genomic reticulation, as evidenced by the correlation between CFs and the amount of interspecific gene flow in simulated data sets. For example, the true clade (D, (C, C*_i_*)) was recovered with CFs of 0.99, 0.87, and 0.12 for the low, medium, and strong gene flow data sets, respectively. In the strong gene flow data set, an erroneous clade composed of the two reticulating taxa (B*_i_*, C*_i_*) is present in the primary concordance tree with a CF of 0.75. Population trees matched the primary concordance tree topologies for all three simulated data sets. In all cases the value of *α* had little effect on the results.

Primary concordance trees inferred from the empirical data recovered a wide range of CFs, with some clades having high support, and others showing evidence of reticulation. In all cases, the superba clade (*P. cyathophylloides*, (*P. cyathophylla*, *P. superba*)) was well-resolved with significant CFs (Fig. 4c,d). However, conflicting relationships in this clade were also evident: the clade (*P. cyathophylla*, *P. cyathophylloides*) had a CF of 0.21, and the clade (*P. superba*, *P. cyathophylloides*) had a CF of 0.07. These results were independent of *α*. The asymmetry in CF values suggests that reticulation, as opposed to incomplete lineage sorting alone, may have occurred. This comparison is in principle analogous to the D-statistic (Ané 2010; Chung and Ané 2011), utilizing gene tree heterogeneity as opposed to allele frequencies.

The value of *α* had a greater effect on results in the rex-thamnophila clade (Fig. 4c,d), where conflict was more evident. When *α* was low (0.1), the only clade with significant support grouped the two sampled populations of *P. rex* subsp. *rex*. In other clades found with low support, *P. thamnophila* was either monophyletic or paraphyletic, with *P. thamnophila* subsp. *thamnophila* grouping with either *P. rex* subsp. *rex* or *P. rex* subsp. *lipskyana*. When *α* was high (100), the monophyly of *P. thamnophila* was significantly supported (Fig. 4d), and relationships among the three subspecies of *P. rex* remained unresolved.

In contrast to the simulation results, population trees of *Pedicularis* sect. *Cyathophora* did not match the primary concordance trees. Most notably, the population trees matched the ML trees in that *P. thamnophila* was not monophyletic, with *P. thamnophila* subsp. *thamnophila* consistently being placed as sister to *P. rex* subsp. *rex* (Fig. 4e,f), and *P. thamnophila* subsp. *cupuliformes* being placed as sister to the rest of the rex-thamnophila clade.

### Tests for Introgression

*D-statistic tests: simulated data.—*The four-taxon D-statistic test accurately detected introgression in all simulations where it occurred (tests 1.1 and 1.2; Table 2), and rejected introgression where it did not (tests 2.1–2.10). In some cases, however, significant introgression was falsely detected (3.1–3.10). These include tests where P3 shares fewer derived alleles with P1 relative to P2 because P1 received introgressed DNA from a more distant clade (tests 3.1–3.3). As an example, taxon A shares more derived alleles with taxon B relative to B*_i_* in test 3.1 not as a consequence of having introgressed with B, but rather because B*_i_* received alleles from the more distant taxon C*_i_*. A false positive is also detected when the P3 taxon is a close relative of the true source of introgression (C*_i_*), such that the signal picked up is merely the proportion of P3’s genome that is shared with C*_i_* through common ancestry (tests 3.4–3.7). While these do not represent true false positives in the sense that introgression did actually occur from a lineage that P3 is a member of, it is false in the sense that introgression is still detected whether or not the true hybridizing taxon is sampled; thus, the test would lead to the conclusion that both P3 taxa had experienced introgression with P2. Finally, tests where B*_i_* is in the P3 position (tests 3.8–3.10), such that introgression is being tested in the opposite direction it actually occurred, also yielded a positive result.

**Table 2:** Patterson’s four-taxon D-statistic measure of introgression presented as a *Z*-score, applied to RAD sequences simulated under the “weak gene flow” scenario, where unidirectional introgression occurred from taxon C*_i_* into B*_i_* as shown in Figure 2; some tests do not include both taxa

Test	P1	P2	P3	O	Z
1.1	A	B*_i_*	C*_i_*	o	**11.34**
1.2	B	B*_i_*	C*_i_*	o	**14.47**
2.1	A	B	C*_i_*	o	0.26
2.2	A	B	C	o	0.03
2.3	A	B	D	o	0.56
2.4	C	D	A	o	1.66
2.5	C	D	B	o	1.01
2.6	C	C*_i_*	A	o	0.58
2.7	C	C*_i_*	B	o	0.36
2.8	C	C*_i_*	D	o	0.44
2.9	C	C*_i_*	D	A	0.50
2.10	C	C*_i_*	D	B	0.20
3.1	B*_i_*	B	A	o	**7.30**
3.2	B*_i_*	B	A	D	**13.73**
3.3	B*_i_*	B	A	C	**15.43**
3.4	B	B*_i_*	C	o	**15.88**
3.5	A	B*_i_*	C	o	**9.96**
3.6	B	B*_i_*	D	o	**8.74**
3.7	A	B*_i_*	D	o	**5.48**
3.8	D	C	B*_i_*	o	**4.38**
3.9	D	C*_i_*	B*_i_*	o	3.08
3.10	C	C*_i_*	B*_i_*	o	**5.87**

Note: In each test, taxa are arranged such that the ABBA pattern is more frequent than BABA. Significant results are in bold.

The partitioned D-statistic test, in contrast, accurately detected the presence or absence of introgression in all simulated data sets, including those scenarios which led to false positives in the four-taxon test. When introgression occurred only from P3_1_ and not P3_2_, a significant value of D_1_ and non-significant value of D_2_ were correctly obtained (tests 4.1–4.4; Table 3). In the multiple-reticulation data set, where introgression occurred from both P3 lineages independently, both D_1_ and D_2_ were significant (tests 4.3 and 4.5). These results highlight the importance of sampling multiple lineages in order to more precisely identify introgressing taxa. For example, if C*_i_* had not been sampled, such that C was the only representative of (C, C*_i_*), then the partitioned D-statistic test would indicate that C introgressed with B*_i_* (tests 4.1 and 4.2). Only by having more sampled lineages in (C, C*_i_*) are we able to repeat the test at more shallow nodes in the phylogeny (tests 4.3 and 4.5), which yields the results that introgression occurred only from taxon C*_i_* since its divergence from C.

**Table 3:** Partitioned D-statistic test for introgression performed on the “weak gene flow” and “multiple-reticulation” simulated data sets

Test	P1	P2	P3_1_	P3_2_	O	C*_i_* into B*_i_*			C and C*_i_* into B*_i_*		
						*Z*_12_	*Z*_1_	*Z*_2_	*Z*_12_	*Z*_1_	*Z*_2_
4.1	B	B*_i_*	C	D	o	**8.71**	**12.62**	1.40	**18.27**	**30.90**	1.66
4.2	A	B*_i_*	C	D	o	**6.28**	**9.31**	1.37	**15.39**	**22.30**	1.96
4.3	B	B*_i_*	C*_i_*	C	o	**12.39**	**7.66**	0.68	**23.97**	**11.10**	**10.31**
4.4	B	B*_i_*	C*_i_*	D	o	**9.35**	**13.89**	0.71	**18.07**	**33.90**	1.63
4.5	A	B*_i_*	C*_i_*	C	o	**10.17**	**6.64**	0.32	**22.68**	**5.11**	**6.56**
4.6	A	B*_i_*	C*_i_*	D	o	**6.53**	**11.74**	1.61	**15.41**	**21.82**	2.13
5.1	A	B	C	D	o	0.32	0.52	2.04	2.56	0.84	0.97
5.2	A	B	C*_i_*	C	o	0.27	0.11	0.93	2.89	0.92	0.32
5.3	A	B	C*_i_*	D	o	0.17	0.26	1.86	2.43	0.83	1.24
5.4	C	D	B	A	o	1.47	0.47	1.06	1.26	0.54	1.23
5.5	D	C*_i_*	B	A	o	2.07	0.79	1.31	0.50	0.12	1.96
6.1	C	C*_i_*	B*_i_*	B	o	0.28	**7.84**	0.53	2.50	1.86	1.28
6.2	C	C*_i_*	B*_i_*	A	o	0.20	**6.40**	0.45	2.50	1.85	0.21
6.3	D	C*_i_*	B*_i_*	A	o	1.40	**11.14**	1.68	0.28	**21.09**	2.59
6.4	D	C*_i_*	B*_i_*	B	o	1.23	**14.01**	0.70	0.46	**30.40**	2.06
6.5	D	C	B*_i_*	A	o	1.15	**9.87**	1.27	1.91	**20.90**	1.90
6.6	D	C	B*_i_*	B	o	1.01	**11.70**	0.77	1.99	**32.25**	1.08
7.1	B	B*_i_*	A	C*_i_*	o	0.29	**9.68**	**16.06**	0.58	**21.22**	**35.54**
7.2	B	B*_i_*	A	C	o	0.57	**9.68**	**16.31**	0.63	**21.79**	**33.54**
7.3	C	C*_i_*	D	B*_i_*	o	0.91	1.35	**3.65**	2.01	0.74	2.21
7.4	C	C*_i_*	D	B	o	0.27	1.23	1.22	2.17	0.47	1.85

Note: Results are presented as *Z*-scores for D_12_, D_1_, and D_2_, respectively. As in Table 2, some tests do not include all taxa participating in introgression. In each row, taxa are arranged such that the dominant pattern indicates introgression into P2 (i.e., A B _ _ A). Significant results are in bold.

The partitioned D-statistics accurately rejected introgression in tests where it did not occur (tests 5.1–5.5), and correctly indicated the direction of introgression when it did occur. For example, a significant D_1_ was detected when B*_i_*, the recipient of introgressed DNA, was tested in the P3 position (tests 6.1–6.6). However, because the introgressed alleles in these cases are unique to the P3_1_ lineage (B*_i_*), having come from P2 (C*_i_*), they are not shared between multiple members of the P3 clade by ancestry (e.g., between B*_i_* and A or B). Thus, the value of D_12_ does not deviate significantly from 0.

*Four-taxon D-statistic tests: empirical data.—*Uncertainty in the topology of the rex-thamnophila clade arising from the BUCKy analysis yielded a large number of distinct four-taxon subtrees on which to perform D-statistic tests. We present selected results from these tests below, with the full list available in online Appendix 3. Test results are reported as ranges of *Z*-scores from multiple replicates, where a single replicate constitutes a unique sampling of redundant individuals within taxa for a given subtree. The number of RAD loci for which data were available across all four taxa in a test ranged from about 5000 to 21,000, of which 1%–5% contained at least one informative discordant site. More loci were available in tests performed among closer relatives.

We first tested whether introgression occurred between the rex-thamnophila and superba clades, using *P. przewalskii* as the outgroup. These yielded no evidence of introgression (tests 8.1–8.6; Table 4). Next, we tested for introgression within each clade, using members of the other clade as the outgroup. No significant results were detected between any members of the superba clade (tests 9.1–9.3). The two samples of *P. cyathophylloides* have insufficient differences to detect introgression into one versus the other (tests 9.2 and 9.3). *Pedicularis cyathophylloides* shares more derived alleles with *P. cyathophylla* than with *P. superba* (test 9.1), consistent with the CF results, which showed a greater CF for (*P. cyathophylloides*+*P. cyathophylla*) relative to (*P. cyathophylloides*+*P. superba*). However, the difference is non-significant, suggesting incomplete lineage sorting alone may be sufficient to explain this result.

**Table 4.** Patterson’s four-taxon D-statistic test for introgression in *Pedicularis* sect. *Cyathophora*

Test	P1	P2	P3	O	Range *Z*	*n*Sig/*n*
8.1	C_1_	C_2_	RT	W	N/A	0/14
8.2	Y	CS	RT	W	(0.00, 3.24)	0/42
8.3	S	CY	RT	W	(0.92, 2.77)	0/42
8.4	RT	RT	C	W	(0.06, 2.81)	0/84
8.5	RT	RT	Y	W	(0.06, 2.60)	0/42
8.6	RT	RT	S	W	(0.00, 1.88)	0/42
9.1	S	Y	C	RT	(1.35, 3.03)	0/14
9.2	C_1_	C_2_	Y	RT	N/A	0/7
9.3	C_1_	C_2_	S	RT	N/A	0/7
10.1	R	T*_t_*	T*_c_*	CYS	**(5.05, 11.14)**	20/20
10.2	R*_l_*	R*_r_*	T*_c_*	CYS	(0.00, 1.73)	0/16
10.3	R*_o_*	R*_r_*	T*_c_*	CYS	**(2.84, 5.06)**	8/8
10.4	R*_o_*	R*_l_*	T*_c_*	CYS	(1.43, 3.04)	0/8
10.5	R*_l_*	R*_r_*	T*_t_*	CYS	(1.52, 2.96)	0/16
10.6	R*_o_*	R*_r_*	T*_t_*	CYS	**(7.15, 8.65)**	8/8
10.7	R*_o_*	R*_l_*	T*_t_*	CYS	**(4.26, 6.20)**	8/8
10.8	T*_c_*	T*_t_*	R*_o_*	CYS	**(8.96, 11.20)**	4/4
10.9	T*_c_*	T*_t_*	R*_r_*	CYS	**(9.48, 12.07)**	4/4
10.10	T*_c_*	T*_t_*	R*_l_*	CYS	**(8.92, 11.79)**	4/4

Note: Each test was repeated over all possible four-sample replicates (*n*), with a range of *Z*-scores reported, and the number of significant replicates shown (*n*Sig). Taxa are identified by codes listed in Table 1, with numeric subscripts distinguishing individual samples. When no subscript is given, test replicates include all individuals sampled (e.g., R*_r_* = R*_r_*_1_ or R*_r_*_2_; CS = C_1_ or C_2_ or S). In each row, taxa are arranged such that the dominant pattern is always ABBA.

In contrast, within the rex-thamnophila clade nearly all individuals showed significant evidence of introgression. Given a test topology in which the two subspecies of *P. thamnophila* are paraphyletic in positions P3 and P2, *P. thamnophila* subsp. *cupuliformes* showed significant introgression with *P. thamnophila* subsp. *thamnophila* relative to any sample of *P. rex* in position P1 (test 10.1). Testing each subspecies of *P. thamnophila* separately in position P3, with two samples from *P. rex* in positions P1 and P2, we found that *P. thamnophila* subsp. *cupuliformes* may have introgressed with *P. rex* subsp. *rex* relative to *P. rex* subsp. *rockii* (test 10.3), while *P. thamnophila* subsp. *thamnophila* appears to have introgressed with both *P. rex* subsp. *rex* and *P. rex* subsp. *lipskyana* relative to *P. rex* subsp. *rockii* (tests 10.6 and 10.7). Neither subspecies of *P. thamnophila* showed greater introgression with *P. rex* subsp. *rex* relative to *P. rex* subsp. *lipskyana* (tests 10.2 and 10.5).

Given a topology in which the two subspecies of *P. thamnophila* are monophyletic, occupying positions P1 and P2, tests placing each subspecies of *P. rex* in position P3 showed very strong introgression with *P. thamnophila* subsp. *thamnophila* relative to *P. thamnophila* subsp. *cupuliformes* (tests 10.8–10.10).

*Partitioned D-statistic tests: empirical data.—*The partitioned D-statistic test could only be performed using RAD loci containing sites with incongruent allele patterns across the five taxa being tested. Fewer sites met this criterion than for the four-taxon test, so statistical power was comparatively limited. In some cases, fewer than 100 sites for each allele pattern were available (online Appendix 3). In contrast to the four-taxon test, which is agnostic about the directionality of introgression, the partitioned D-statistic allows the direction to be explictly tested.

Treating the two subspecies of *P. thamnophila* as monophyletic in positions P3_1_ and P3_2_, test 11.1 (Table 5) suggests introgression may have occurred between the *P. thamnophila* clade and *P. rex* subsp. *rex* relative to *P. rex* subsp. *rockii*, as evidenced by a significant D_12_. However, D_1_ was significant in only one of eight replicates, and D_2_ was consistently non-significant, suggesting that introgression did not occur from either subspecies of *P. thamnophila* since their divergence from each other. This result could be interpreted in one of two ways. First, introgression may have occurred predominantly from *P. rex* subsp. *rex* into both subspecies of *P. thamnophila* (or their ancestor). This would explain how the latter both exhibit alleles that are derived in *P. rex* subsp. *rex* relative to *P. rex* subsp. *rockii*, but *P. rex* subsp. *rex* does not to contain any alleles derived uniquely in either subspecies of *P. thamnophila*. Alternatively, introgression may have occurred into *P. rex* subsp. *rex* from an unsampled lineage which diverged from the ancestor of the two sampled *P. thamnophila* subspecies, or from their direct ancestor if it occurred before their divergence. Either scenario would yield a significant D_12_ but non-significant D_1_ and D_2_. Additional sampling of *P. thamnophila* will be necessary to further clarify this result. Tests 11.2 and 11.3 similarly show non-significant or weak signals of introgression from *P. thamnophila* into the other subspecies of *P. rex*.

**Table 5.** Partitioned D-statistic test for introgression in *Pedicularis* sect. *Cyathophora*

Test	P1	P2	P3_1_	P3_2_	O	P3_1_ and P3_2_		P3_1_ only		P3_2_ only	
						*Z*_12_	*n*Sig/*n*	*Z*_1_	*n*Sig/*n*	*Z*_2_	*N*sig/*n*
11.1	R*_o_*	R*_r_*	T*_t_*	T*_c_*	CYS	**(3.31, 5.80)**	8/8	(1.12, **3.71**)	1/8	(0.09, 2.29)	0/8
11.2	R*_o_*	R*_l_*	T*_t_*	T*_c_*	CYS	(1.52, 2.78)	0/8	(2.19, **3.74**)	2/8	(0.33, 2.15)	0/8
11.3	R*_r_*	R*_l_*	T*_t_*	T*_c_*	CYS	(0.00, 2.44)	0/16	(0.07, 1.29)	0/16	(0.24, 1.98)	0/16
12.1	T*_c_*	T*_t_*	R*_o_*	R*_r_*	CYS	**(7.54, 10.90)**	8/8	**(3.31, 5.65)**	8/8	**(5.00, 7.51)**	8/8
12.2	T*_c_*	T*_t_*	R*_o_*	R*_l_*	CYS	**(6.91, 10.99)**	8/8	(2.56, **5.24**)	7/8	**(4.22, 6.77)**	8/8
12.3	T*_c_*	T*_t_*	R*_r_*	R*_l_*	CYS	**(8.03, 10.99)**	16/16	**(4.64, 7.77)**	16/16	**(3.45, 6.84)**	16/16
12.4	T*_c_*	T*_t_*	R*_r_*_1_	R*_r_*_2_	CYS	**(10.60, 11.75)**	4/4	(1.88, 2.83)	0/4	**(3.17, 4.05)**	4/4
12.5	T*_c_*	T*_t_*	R*_l_*_1_	R*_l_*_2_	CYS	**(7.93, 11.15)**	4/4	(0.44, 1.18)	0/4	**(3.25, 5.44)**	4/4
13.1	R*_r_*_2_	R*_r_*_1_	R*_l_*	T*_t_*	CYS	(0.18, 0.85)	0/8	(0.27, 2.73)	0/8	(0.19, 1.28)	0/8
13.2	R*_l_*_2_	R*_l_*_1_	R*_r_*	T*_t_*	CYS	(0.18, 1.19)	0/8	(0.23, 1.57)	0/8	(0.20, 1.24)	0/8
13.3	R*_r_*_2_	R*_r_*_1_	R*_o_*	T*_t_*	CYS	(0.41, 1.13)	0/4	(0.07, 1.37)	0/4	(1.15, 2.30)	0/4
13.4	R*_l_*_2_	R*_l_*_1_	R*_o_*	T*_t_*	CYS	(0.30, 0.94)	0/4	(0.25, 0.85)	0/4	(1.17, 1.89)	0/4

Note: Each test was repeated over all possible five-taxon subtree replicates (*n*) to yield a range of *Z*-scores. The number of significant replicates is given by *n*Sig. Taxa are identified using codes following Table 4. *Z*-scores are reported for each respective D-statistic, representing asymmetry in incongruent allele patterns for which the derived allele is shared by both P3_1_ and P3_2_ (Z_12_), by P3_1_ but not P3_2_ (Z_1_), or by P3_2_ but not P3_1_ (Z_2_). Tests are arranged such that the dominant pattern is always introgression into P2 (i.e., A B _ _ A).

For all tests placing subspecies of *P. rex* in positions P3_1_ and P3_2_, significant introgression was detected into *P. thamnophila* subsp. *thamnophila* when tested relative to *P. thamnophila* subsp. *cupuliformes* (tests 12.1–12.3). Moreover, in addition to a very significant D_12_, all tests have a significant D_1_ and D_2_, suggesting that all three subspecies of *P. rex* have introgressed into *P. thamnophila* subsp. *thamnophila* independently since their divergences from one another.

Focusing closer to the tips of the phylogeny, and considering the test placing the two samples of *P. rex* subsp. *rex* in the P3 clade (and therefore testing for introgression occurring after their divergence from each other), we find significant introgression from only one sample of *P. rex* subsp. *rex* into *P. thamnophila* subsp. *thamnophila*, suggesting this event occurred very recently (test 12.4). Similarly, introgression also occurred from one of the two samples of *P. rex* subsp. *lipskyana* since their even more recent divergence (test 12.5). In both cases, the introgressing population is the one located geographically closer to the sampled population of *P. thamnophila* subsp. *thamnophila*. (“Rr_2_” and “Rl_2_”; Fig. 1).

*The proportion of genomic introgression.—*In simulated data, the mean proportion of genomic introgression from C*_i_* into B*_i_* was accurately estimated to be 1%, 10%, and 90% for the weak, medium, and strong gene flow data sets, respectively, corresponding with the simulation parameters. However, as with the four-taxon D-statistic, we find this test is similarly biased by shared ancestry. For example, recall that in these simulations, taxon C did not itself introgress with B*_i_*, but was a close relative of the taxon that did, C*_i_*; yet the mean proportion of genomic introgression from C into B*_i_* was estimated to be 0.09%, 8.7%, and 87%. Moreover, in the multiple-reticulation data set, which includes two taxa (C and C*_i_*) independently introgressing into B*_i_* at the same rate as in the weak gene flow data set (0.01), we measured the mean proportions of genomic introgression from C and C*_i_* each to be 0.02, twice the expected value, showing an additive effect of introgression from multiple closely related lineages.

Using this same method, we calculated the proportion of genomic introgression among members of the rex-thamnophila clade (online Appendix 3). Genomic introgression from *P. rex* subsp. *rex* into *P. thamnophila* subsp. *thamnophila* is estimated to be 26.6%, and that from *P. rex* subsp. *lipskyana* into *P. thamnophila* subsp. *thamnophila* as 8.7%. Although this is meant to provide a minimum estimate, the additive nature of this measurement when hybridization occurs from multiple taxa, coupled with our results showing that all three subspecies of *P. rex* introgressed into *P. thamnophila* subsp. *thamnophila* independently, leads us to suspect these estimates are inflated.

### Detecting Errors in Tree Reconstruction Due to Introgression

*Partitioned D-statistic.—*In the simulated data sets the signal of introgression measured by D_12_ proved an accurate indicator of monophyly versus paraphyly of taxa P3_1_ and P3_2_, by detecting a non-significant D_12_ in all cases for which paraphyletic species were grouped as a P3 clade (tests 7.1–7.4). In the empirical data, a significant D_12_ was detected in some but not all tests involving the two subspecies of *P. thamnophila* in the P3 position. However, as we noted above, this signal could have been caused by introgression in the opposite direction. Alternative topologies where *P. thamnophila* subsp. *thamnophila* is nested within *P. rex* received no support, all having non-significant D_12_ values (tests 13.1–13.4).

**Figure 5:**
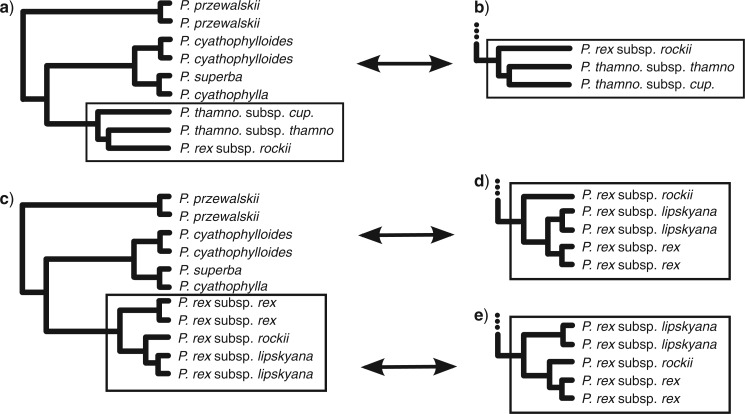
Censored data sets, in which one or more taxa have been removed to minimize the effect of their introgressed alleles, are used to compare alternative topologies within the Shimodeira–Hasegawa test. The ML topology with *P. rex* subsp. *rex* and *P. rex* subsp. *lipskyana* removed a) is compared with an alternative topology in which *P. thamnophila* is monophyletic b) Similarly, the ML topology with both subspecies of *P. thamnophila* removed c) is compared with two alternative resolutions for the three subspecies of *P. rex* d) and e).

*Censored comparisons of alternative topologies.—*With complete taxon sampling, the SH test shows significant support for the unconstrained ML topology in which *P. thamnophila* is paraphyletic, compared to the constrained topology in which it is monophyletic (Δ*ln*L = 198.90*,p* < 0.05). However, after removing *P. rex* subsp. *rex* and *P. rex* subsp. *lipskyana*, leaving only *P. rex* subsp. *rockii* (Fig. 5a,b), monophyly of *P. thamnophila* is favored, though the unconstrained topology cannot be significantly rejected (Δ*ln*L = 85.82*,p* > 0.05). Applying the same approach reveals the effect of selective taxon removal on relationships within *P. rex*. With complete sampling, the ML topology ((*P. rex* subsp. *rockii*, *P. rex* subsp. *lipskyana*), *P. rex* subsp. *rex*) was favored over a constrained topology ((*P. rex* subsp. *rex*, *P. rex* subsp. *lipskyana*), *P. rex* subsp. *rockii*) (Δ*ln*L = 420.22*,p* > 0.05). After removing each subspecies of *P. thamnophila* from the data set (Fig. 5c,e), the constrained topology in which *P. rex* subsp. *rockii* is sister to the other two subspecies is a significantly better fit to the data than the two alternatives (Δ*ln*L = 240.60,252.89;*p* < 0.01).

## Discussion

### Phylogenetic Inference using RADseq Data

In this study we use RADseq data to resolve shallow phylogenetic relationships and test for introgression in a systematically problematic clade of flowering plants, *Pedicularis* sect. *Cyathophora*. In comparison to previous studies, which used sequence data sets of at most a few markers, each on the order of 1 kbp in length, the RADseq data set is notable for its size, having more than 40,000 loci and 60,000 potentially informative sites in the largest supermatrix. As workflows for processing RADseq data for phylogenetic analysis are not yet widely available, we developed new software (pyRAD) for this purpose.

As sequencing technology improves and becomes more cost-effective, reduced-representation genomic data sets are rapidly becoming attainable for non-model organisms; indeed, since we began this study up to 4X the output can now be produced for a similar cost, allowing greater multiplexing of individuals and deeper coverage. In this context, the question of how such data can be used to reconstruct the tree of life comes increasingly to the fore. A common approach applies the principle of total evidence, namely that phylogeny should be reconstructed from as much data as possible, with the aim of identifying the dominant signal (e.g., Kluge 1989). A contrasting approach asserts that phylogenetic signal should be investigated on a gene-by-gene basis, with the causes of gene tree incongruence in mind (e.g., Rannala and Yang 2008). RADseq data are easily concatenated for use in total-evidence tree inference, as demonstrated in our ML supermatrix analyses. However, the data are less suited for gene tree approaches such as concordance analysis, because individual RAD loci as assembled here are limited in sequence length, contain relatively few variable sites, and do not generally yield resolved gene trees. As a result, a large proportion of loci are necessarily excluded from consideration, and selectively choosing the most variable RAD loci poses a risk of introducing potential biases; for example, if these regions are more variable as a result of retaining ancestral polymorphisms or by more frequently representing regions of introgressed DNA.

Improvements in next-generation sequencing methods have the potential to greatly improve the utility of RADseq and related approaches to phylogenetic studies. Among these, paired-end Illumina sequencing may yield the most immediate and significant benefits. For example, it could double the length of reads produced using GBS methods (in which both ends of a DNA fragment contain restriction enzyme recognition sites), yielding 200–300 bp of sequence data per locus. For RADseq protocols, in which fragment size selection is performed by random shearing, even greater sequence lengths are achievable, because long contigs can be assembled from partially overlapping sheared-end reads. For example, (Etter et al. 2011) used this method to assemble contigs up to several hundred bp in length.

### Detecting Introgression

Using simulations, we showed that on a four-taxon subtree, Patterson’s D-statistic test can have a high rate of type-1 error in detecting introgression between taxa in positions P2 and P3, because it does not discriminate between incongruent allele patterns that arise directly from hybridization of the specific taxa sampled in the test, and patterns that would arise if hybridization had occurred between one of the sampled taxa and a close relative of the other. Given a taxon in position P2 and multiple candidate taxa in position P3, the partitioned D-statistic test can be used to more precisely identify which of the P3 taxa contributed to introgression, under the assumption that the two P3 candidates have not exchanged genes with each other. By distinguishing between introgressed alleles that are unique to individual taxa in P3 and those that are shared by common ancestry, the test can reveal the timing of introgression relative to phylogenetic divergence events in the P3 clade. The partitioned D-statistic test is thus a novel extension of the method that improves its utility above the species level.

While D-statistic tests were originally applied to test ancient admixture between now extinct and modern human populations (Green et al. 2010; Skoglund and Jakobsson 2011; Meyer et al. 2012) they have more recently been applied at deeper phylogenetic scales and within more diverse clades (The Heliconius Genome Consortium 2012), where teasing apart introgression from the signal of shared ancestry is of increased importance. This is especially true when the P3 lineage contains multiple distinct species or eco-morphs. A failure to distinguish whether introgression occurred independently from each P3 taxon following their divergence from each other could yield false positives that would ultimately inflate estimates of the frequency of natural hybridization.

Since its original description other extensions of the D-statistic methodology have also been developed. In particular, (Meyer et al. 2012) described an “enhanced D-statistic”, which involves performing the four-taxon D-statistic across only a subset of sites for which multiple sampled individuals of the P1 taxon—in their case 30 human populations from sub-Saharan Africa—are all fixed for the ancestral allele. By effectively removing sites where the derived allele differs between P1 and P2 due to sorting of ancestral polymorphisms, this method increases the signal to noise ratio, enhancing the signal of introgression. In contrast to the partitioned D-statistic which aims to improve the performance of D-statistics at deeper phylogenetic scales, the enhanced test is most effective at shallow scales, such as among recently diverged populations, where ancestral polymorphisms are common.

Meyer et al. (2012) similarly described new methods for estimating the proportion of introgressed DNA between groups, including under scenarios where multiple P3 taxa could serve as the source of introgression. Their method is specifically tailored to the Neanderthal and Denisovan data sets, however, where other historical information allows assumptions about the order in which gene flow is likely to have occurred. In other words, they assume Neanderthal gene flow occurred first into all non-African populations, and thus Denisovan gene flow can be detected by measuring the excess signal of archaic ancestry over that expected to be present in all humans outside of Africa. This method may not be suitable to all data sets, and we propose that the partitioned D-statistic provides a more simple and general test to distinguish introgression events from among multiple P3 taxa.

All previous studies applying the D-statistic have utilized a reference genome, which provides linkage information and longer stretches of DNA from which to measure variation in the distribution of incongruent sites. To measure sampling error of D-statistics, as well as their significance, the asymmetry in incongruent allele patterns is generally assessed through a block jack-knife approach (Green et al. 2010), splitting the genome into a set number of blocks and removing them sequentially. Without access to a full genome alignment or other linkage information, we implemented a modification on this strategy based on the theoretical distribution of RADseq data: to the extent RAD loci represent a random distribution of unlinked markers from across the genome, bootstrap re-sampling should provide an accurate measure of the genome-wide variation in incongruence. Previous studies reported no significant effect of jack-knife block size on D-statistic results (Meyer et al. 2012), and our implementation of the bootstrap method to simulated RADseq data accurately detected introgression. Because short-read *de-novo* RADseq loci, such as we use here, appear sufficient for detecting genome-wide patterns of introgression, the application of D-statistic tests could be expanded more broadly, including within diverse groups or organisms that yet lack a reference genome or linkage map. The simulations presented here are limited in scope, however, and further studies will be needed to evaluate how different methods and data types affect D-statistic results.

Analyses of D-statistics across a range of four-and five-taxon subtrees of *Pedicularis* sect. *Cyathophora* revealed clear evidence of recurrent introgression among taxa in the rex-thamnophila clade, with all sampled subspecies of *P. rex* appearing to have exchanged genes at some point with *P. thamnophila* subsp. *thamnophila* relative to *P. thamnophila* subsp. *cupuliformes*. The partitioned D-statistic test showed that for both *P. rex* subsp. *rex* and *P. rex* subsp. *lipskyana*, only one of the two populations sampled in each case yielded a signal of introgression. This suggests that these most recent introgression events occurred since the divergence of the two populations of each subspecies, and may have been localized to particular geographic locations (discussed in more detail below).

### The Effect of Introgression on Phylogenetic Inference

**Figure 6:**
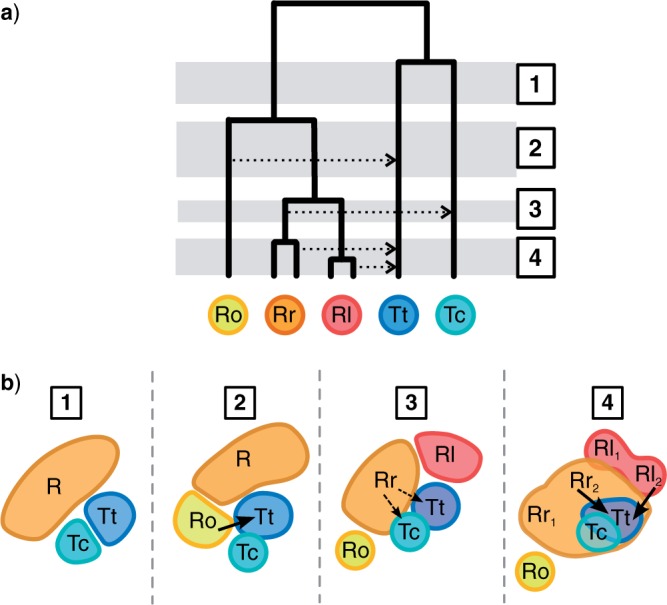
Temporal and geographic reconstructions of population divergence and gene flow, as inferred from the partitioned D-statistic. Codes for taxon names are listed in Table 1. a) Phylogeny of rex-thamnophila based on censored subtree comparisons which account for hybrid introgression. Gray bars indicate distinct time periods discussed in the text, and arrows are the direction of introgressive gene flow. b) Schematic reconstruction of historical biogeography in the rex-thamnophila clade, inferred from present-day distributions and patterns of historical introgression. Overlapping edges indicate geographic overlap, and arrows represent introgression.

Detecting introgression using the D-statistic presents something of a paradox, in that some knowledge of the “true” phylogeny—minimally, a pectinate four-taxon subtree—is required to formulate a hypothesis, while the process of introgression itself acts to obscure those relationships. This motivated us to explore alternative means of assessing whether phylogenetic inference might be positively misled by reticulation events. Using the SH test, we compared the ML total-evidence phylogeny of *Pedicularis* sect. *Cyathophora* with alternative topologies before and after removing selected taxa in order to effectively eliminate the influence of introgressed alleles on tree inference. This revealed two cases in which the total-evidence topology could be inaccurate: first, the paraphyly of *P. thamnophila* (Fig. 4a,b), and second, the position of *P. rex* subsp. *rockii* as sister to *P. rex* subsp. *lipskyana*. Censored SH tests show that, if the signal of introgression is removed, the most likely topology has *P. thamnophila* being monophyletic, and *P. rex* subsp. *rockii* as sister to the other subspecies of *P. rex* (Fig. 6a).

If this tree is correct, such that *P. thamnophila* subsp. *thamnophila* and *P. thamnophila* subsp. *cupuliformes* do share a most recent common ancestor, a signal of this ancestor may be preserved in introgressed alleles. This idea stems from our simulation results, and makes sense logically under a unidirectional gene flow model, where a significant D_12_ would be detected if the two P3 samples shared a unique history over which share derived alleles could arise. In support of *P. thamnophila* monophyly, tests treating the two subspecies as monophyletic detected a significant D_12_ showing introgression into *P. rex* subsp. *rex* (test 11.1); whereas no alternative tests grouping *P. thamnophila* subsp. *thamnophila* with a *P. rex* taxa recovered a significant D_12_ (tests 13.1–13.4). Further studies are needed to investigate the situations under which D_12_ is informative about ancestral relationships, including complex scenarios including multiple pairs of introgressing taxa, and bi-directional gene flow.

### Temporal and Spatial Sequences of Gene Flow

Of the many factors that may influence the likelihood of gene flow between populations, geographic proximity is among the most important (Jenkins et al. 2010). The phylogenetic signal of introgression is thus potentially informative about historical biogeography to the extent that it reflects ancestral contact zones of geographic ranges. With this in mind, we can approach the results of our investigation of *Pedicularis* sect. *Cyathophora* with the objective of piecing together the temporal and spatial sequences of introgression, geographic isolation, and lineage divergence, which we present as a hypothetical reconstruction (Fig. 6b).

*P. rex* subsp. *rockii* is of particular interest from this perspective. Its geographic range is currently isolated to the south of all other lineages in the rex-thamnophila clade (Figs. 1 and Fig. 6b). Results from censored SH tests and partitioned D-statistic tests indicate that it was the first to branch from the ancestral *P. rex* lineage, and that it introgressed into *P. thamnophila* subsp. *thamnophila*. This suggests that before *P. rex* subsp. *rockii* became geographically isolated, the ranges of these taxa were in contact. Given that *P. rex* subsp. *rockii* shows no evidence of gene flow with either *P. rex* subsp. *rex* or *P. rex* subsp. *lipskyana*, it seems likely that *P. rex* subsp. *rockii* became geographically isolated before the others diverged, that is, during time 2 in Fig. 6, and thus gene flow into *P. thamnophila* subsp. *thamnophila* likely occured during this time.

The next introgression event we identified with confidence, occurring into *P. thamnophila* subsp. *thamnophila* from *P. rex* subsp. *rex* and *P. rex* subsp. *lipskyana*, is more difficult to date precisely. From our data, we can typically only detect the most recent introgression events, which in this case appear to have occurred very recently (time 4), following the divergence of sampled populations within each subspecies of *P. rex*. In both cases, introgression occurred from the geographically more proximate population to the *P. thamnophila* subsp. *thamnophila* sample. This does not preclude, however, one or more earlier hybridization events, which we cannot detect without additional samples from each clade to use for comparison. If gene flow indeed occurred very recently, it is interesting to note that *P. rex* subsp. *lipskyana* does not currently occur sympatrically with *P. thamnophila* subsp. *thamnophila* (time 4; Fig. 6b). This suggests that one or both of these taxa underwent a recent range contraction since their hybridization.

An additional gene flow event may have occurred from *P. rex* subsp. *rex* into either both subspecies of *P. thamnophila*, or perhaps into only *P. thamnophila* subsp. *cupuliformes*, at some point after time 3. This would seem the most parsimonious explanation for the presence of alleles derived in *P. rex* subsp. *rex* relative to *P. rex* subsp. *rockii* being present in both subspecies of *P. thamnophila*, yielding a significant D_12_, while neither subspecies of *P. thamnophila* appears to have uniquely derived alleles, relative to each other, introgressed into *P. rex* subsp. *rex*. In interpreting this result we are limited by having only two samples from *P. thamnophila*. Because D-statistics are a comparative measurement, showing only whether gene flow occurred into one taxon more so than into another, it is difficult to determine whether the taxon which received less gene flow in fact received any at all, unless there is another sample which received even less gene flow with which to compare it.

Our clearest result, which holds independently of whether the two subspecies of *P. thamnophila* are monophyletic or paraphyletic, shows that *P. thamnophila* subsp. *thamnophila* exchanged genes with *P. rex*. If *P. thamnophila* is monophyletic, our results can additionally be interpreted to show a pattern of highly asymmetric gene flow, with introgression occurring from *P. rex* much more than in the reverse direction. Such asymmetry is not unexpected in a hybridizing pair when one species is comparatively rare (Levin et al. 1996), as introgressed alleles could spread quickly through a small population, whereas they are more likely to remain localized in a more widespread species. This could be the case among the widespread and common *P. rex* taxa as they hybridized with the narrow endemic *P. thamnophila* taxa. Introgression in this way can even pose a potential extinction risk to the rarer species (Ghosh et al. 2012), particularly if gene flow has only recently been initiated due to a range expansion. With the methods and data used here, it is difficult to ascertain whether introgression occurred persistently through time versus having occurred only recently; however, our results suggest gene flow into *P. thamnophila* subsp. *thamnophila* occurred from several distinct lineages of *P. rex*, including from one which is now geographically isolated and thus likely to have occurred in the past, and two events which are very recent. With these hypotheses in mind, more explicit model-based tests could be used to more accurately infer the timing of divergence and gene flow. This includes isolation-migration models (e.g., IMa2; Hey 2010), or more complex simulation-based models implemented in an approximate Bayesian computation framework (Beaumont 2010).

### Floral Divergence and Isolation

In *Pedicularis* sect. *Cyathophora*, the most conspicuous morphological differences among species are in their flowers, with the majority of variation composing different combinations of three floral characters. These include the length of the corolla tube, which covaries with the presence versus absence of nectar as well as the length that pollen tubes must grow to fertilize the ovules; the length and curvature of the galea (fused upper lobes of corolla) which directs pollen placement onto either the dorsal or ventral side of visiting pollinators; and flower color, which is typically yellow, reddish-purple, white, or some combination thereof (Fig. 1). All species in the superba clade have reddish-purple flowers, but exhibit a wide variety of floral morphologies, one species having a long corolla tube and long-beaked galea (*P. cyathophylla*), one a medium-length corolla tube and short, slightly beaked galea (*P. cyathophylloides*), and the other a short corolla tube and short-beaked galea (*P. superba*). Taxa in the rex-thamnophila clade, in contrast, all have short corolla tubes and short, rounded, beakless galeas. The flowers of *P. thamnophila* are smaller, have a spreading lower corolla lip, and are more consistently yellow, whereas those of *P. rex* are larger, have an adpressed lower lip, and vary in flower color, with yellow, whitish, and reddish-purple forms (the latter characterizing *P. rex* subsp. *lipskyana*).

Floral differences do not seem to closely reflect phylogenetic distances, as taxa in the superba clade exhibit much more differentiated flowers than those in the rex-thamnophila clade. A potential explanation for the great diversity of flowers in *Pedicularis* is reproductive character displacement (Armbruster et al. 1994), particularly, in the way it may occur among the many closely related species which co-occur, flower synchronously, and share pollinators in the Hengduan region. (Eaton et al. 2012) found support for this hypothesis at the community scale, showing both consistent overdispersion (i.e., limiting similarity) of floral traits among co-occurring *Pedicularis* species across local communities, as well as a phylogenetic signal of homoplasy in the evolution of floral traits, suggesting there has been persistent selection driving repeated adaptations to fill available floral niches. A more specific hypothesis, however, is that such selection, rather than being driven by all species of *Pedicularis* that locally co-occur, may instead be caused primarily by interactions among only the most closely related species—those still capable of exchanging genes. (Eaton et al. 2012) found some support for this hypothesis, showing that only the most species-rich communities tend to compose more distantly related species than expected.

This study, in showing different levels of gene flow among taxa in clades exhibiting different degrees of floral differentiation, offers insight into the processes of floral divergence. If differences are driven primarily by selection to reduce interspecific gene flow—the process of reinforcement (Servedio and Noor 2003; Hopkins and Rausher 2012)—then species that experienced past introgression are expected to exhibit more differentiated floral morphologies today. Such a pattern is the opposite of what we observe in *Pedicularis* sect. *Cyathophora*, namely greater gene flow among taxa with more similar flowers. Our results are consistent with a reproductive character displacement scenario in which introgression between taxa inhibits morphological differentiation. This is further supported by the fact that taxa in the superba clade tend to occur at higher elevations and in smaller, more isolated populations, compared to taxa in the rex-thamnophila clade. Following this line of reasoning, floral divergence does not occur during speciation, but rather, taxa which become geographically isolated from close relatives experience greater opportunity for adaptation to local conditions, which in turn sets the stage for further evolution of reproductive isolation.

Several recent studies have shown a contrasting pattern, in which hybridization appears to have played a role in generating phenotypic diversity. In *Heliconius* butterflies, for example, introgression between closely related species has been shown to allow the exchange of supergenes underlying similar mimicry patterns (The Heliconius Genome Consortium 2012); or in Louisiana irises, where hybrids show novel phenotypes with increased fitness relative to their parent species (Arnold et al. 2012). In *Pedicularis*, if hybrid introgression has contributed to adaptive radiation by similarly facilitating the exchange of genes affecting floral phenotypes, it could help explain the high degree of floral diversity and homoplasy in the group (Ree 2005); however, no evidence for this has yet been found.

Central to this explanation is the rate at which reproductive isolating barriers evolve, and the nature of these barriers in *Pedicularis*. Pre-mating isolation facilitated by the differentiation of floral traits can play a significant role in the speciation process, particularly in a clade such as the one studied here, where close relatives capable of hybridization occur sympatrically. In rex-thamnophila, although species exhibit less differentiation in floral traits than in the superba clade, they do exhibit small differences that could have large effect. Pre- and post-zygotic barriers are needed to complete the cessation of gene flow, and in *Pedicularis*, crossing experiments need to be done to provide empirical evidence of current hybridization potential with genomic estimates of past gene flow.

Estimates of hybridization based on morphological determinations indicate that it occurs in as many as 25% of plant species and 10% of animal species (Mallet 2005), but the phenomenon remains rarely investigated and poorly understood in a phylogenetic context. Ideally, phylogenies that accurately represent the history of population divergences could be applied to study patterns of introgression in a comparative framework, where its effects on character evolution and rates of divergence could be inferred. Because interspecific gene flow confounds phylogeny reconstruction, however, such studies remain difficult. In *Pedicularis* sect. *Cyathophora*, introgression was found to significantly affect phylogenetic inference. By applying genome-wide tests for introgression to RADseq data, we were able to identify hybridizing taxa, and to compare specific phylogenetic hypotheses after minimizing the effect of introgressed DNA. This allowed us to recover a new topology not originally supported by any of the phylogenetic inference methods employed, and which was more consistent with geography and morphology. Future analyses, particularly in plants, are likely to benefit from integrating genomic information about hybrid introgression into phylogenetic analyses.

## Supplementary Material

Supplementary material, including data files and/or online-only appendices, can be found at http://datadryad.org and in the Dryad data repository (DOI:10.5061/dryad.bn281).

## Funding

This work was supported by the National Science Foundation [DEB-1119098 to R.H.R. and DEB-1110598 to D.A.R.E.].
